# Relationship between cathepsins and cardiovascular diseases: a Mendelian randomized study

**DOI:** 10.3389/fphar.2024.1370350

**Published:** 2024-07-04

**Authors:** Qiaoqiao Li, Zhongzheng Zhou, Teng Xu, Xueping Gao, Yake Lou, Zijun Chen, Muzi Zhang, Qinghua Fang, Jie Tan, Jing Huang

**Affiliations:** ^1^ Department of Cardiology, The Second Affiliated Hospital of Chongqing Medical University, Chongqing, China; ^2^ Department of Laboratory Medicine, The First Affiliated Hospital of Chongqing Medical University, Chongqing, China

**Keywords:** cathepsins, cardiovascular diseases, Mendelian randomization, causality, single nucleotide polymorphisms

## Abstract

**Background:** Cardiovascular diseases (CVDs) are the leading age-related disorders worldwide, with their prevalence increasing annually. Cathepsins are protein-degrading enzymes essential for processes such as intracellular protein breakdown, apoptosis, and immune responses. Recent studies suggest a potential link between cathepsins and CVDs, yet the exact causal relationship remains to be elucidated. To address this, we propose using Mendelian randomization (MR) to explore the causal relationships between cathepsins and CVDs.

**Methods:** We obtained single nucleotide polymorphism (SNP) data for cathepsins from the INTERVAL study, a publicly accessible genome-wide association study (GWAS) dataset. Outcome SNP data were sourced from seven distinct GWAS datasets, ensuring a comprehensive analysis across multiple cardiovascular outcomes. For MR analysis, we primarily employed the inverse variance weighted (IVW) method, known for its efficiency when all SNPs are valid instruments. This was supplemented by the weighted median and MR-Egger methods to provide robustness against potential violations of MR assumptions, such as pleiotropy. The IVW method offers precision and efficiency, the weighted median method adds robustness against invalid instruments, and the MR-Egger method helps identify and correct for pleiotropic biases. Cochran’s Q test was utilized to assess heterogeneity, and sensitivity analyses were conducted using MR-PRESSO and the leave-one-out approach.

**Results:** The strength of the associations between exposure and outcome was measured using odds ratios (ORs), and results were presented with 95% confidence intervals (CIs). The cathepsin E increases the risk of myocardial infarction (MI) (OR = 1.053%, 95% CI: 1.007–1.101, *p* = 0.024) and ischemic stroke (IS) (OR = 1.06%, 95% CI: 1.019–1.103, *p* = 0.004). Conversely, cathepsin L2 decreases the risk of chronic heart failure (CHF) (OR = 0.922%, 95% CI: 0.859–0.99, *p* = 0.025) and atrial fibrillation (AF) (OR = 0.956%, 95% CI: 0.918–0.996, *p* = 0.033). Cathepsin O was associated with an increased risk of IS (OR = 1.054%, 95% CI: 1.008–1.102, *p* = 0.021) and AF (OR = 1.058%, 95% CI: 1.02–1.098, *p* = 0.002).

**Conclusion:** Our MR analysis reveals that cathepsin E is a risk factor for MI and IS, cathepsin L2 offers protective effects against CHF and AF, and cathepsin O increases the risk for IS and AF.

## Introduction

CVDs are the most common geriatric diseases, affecting 29% of the elderly worldwide, with increasing incidence rate (Fryar et al., 2012). The pathogenesis of CVDs is multifaceted, involving various risk factors and closely linked to cellular protein metabolism (Verbrugge et al., 2015). Cathepsins, which function as lysosomal enzymes, play a pivotal role in protein degradation within the cell’s acidic compartments. Elevated levels of cardiac, aortic, and plasma tissue proteases have been noted in patients with CVDs (Turk et al., 2001). There are 15 types of cathepsins in the human body, namely, Cathepsin A, B, C, D, E, F, G, H, K, L, O, S, L2, W, and Z. Several of these cathepsins act as biomarkers or risk factors for CVDs (Turk and Bode, 1991).

Cathepsins exhibit both pathological and physiological roles within and outside of cells. Beyond their function in degrading endocytic and endogenous proteins, they are also involved in antigen processing and presentation (Riese et al., 1996). The involvement of cathepsins in activating inflammatory molecules, regulating immunity, facilitating cell migration, managing cholesterol metabolism, promoting neovascularization, inducing cell death, signaling cellular processes, and contributing to tissue fibrosis underscores their impact on CVDs (Zhang et al., 2020). Studies have shown that while cathepsins are pathogenic in conditions such as atherosclerosis and abdominal aneurysms, many of the same enzymes also offer cardioprotective effects in hypertension, cardiac hypertrophy, and MI (Qin and Shi, 2011). To assess whether there is a causal relationship between cathepsins and CVDs, we propose employing the MR method. MR is a method that utilizes genetic variations, which are predetermined at conception and associated with specific exposures, to explore causal relationships between these exposures and health outcomes (Emdin et al., 2017). The random allocation of genes at conception serves as a natural experiment, helping to mitigate confounding factors that could otherwise bias the results (Emdin et al., 2017). Additionally, because these genetic variants are fixed before any disease develops, they are unaffected by the disease, thereby minimizing the risk of reverse causality (Sekula et al., 2016).

MR offers significant advantages over basic research methods in establishing causality, primarily by addressing two major limitations in observational studies: 1. Reduction of Confounding: MR uses genetic variants as instrumental variables, which are randomly assigned at conception, to reduce the influence of confounders that often bias observational studies. This approach mimics the randomization of a controlled trial, providing clearer insights into causal relationships (Sheehan et al., 2008). 2. Mitigation of Reverse Causation: Since genetic variants precede the onset of disease, MR ensures the directionality of the relationship from exposure to outcome, avoiding issues of reverse causation. MR also enhances generalizability, allows exploration of biological mechanisms, and is ethically feasible for studying harmful exposures. It complements observational studies, providing robust evidence that strengthens or challenges observed associations. Thus, MR is a powerful tool in epidemiology for confirming and understanding causal relationships where traditional methods may be inadequate (Sheehan et al., 2008).

Another advantage of the MR Approach is that it can provide a link between relevant proteins and disease at the genetic level, such as the article published by Tan JS et al., in 2022 suggesting that genetic susceptibility to anti-cytomegalovirus IgG levels increases the risk of coronary artery disease (Tan et al., 2022). Gao Q et al., published in 2022, also used MR analysis to reveal a link between disorders of lipoprotein and CVDs (Gao et al., 2022). Therefore we also used MR methods to investigate the potential link between cathepsins and CVDs.

## Materials and methods

### Mendelian randomization data and process

**FIGURE 1:**
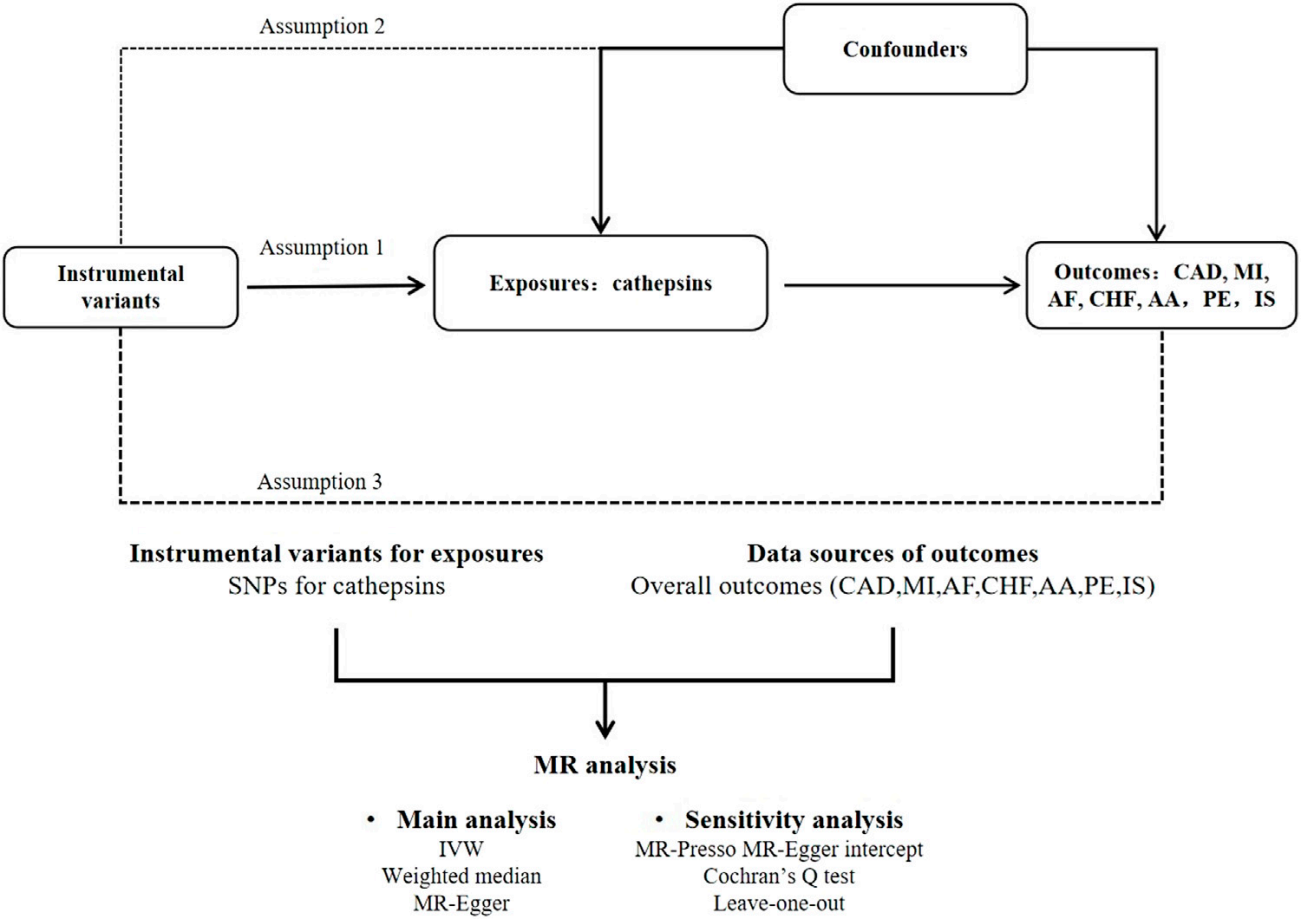
Flow chart of MR analysis. This study adheres to the three core assumptions of MR: Assumption 1: Indicated by the solid line, the instrumental variants directly influence the incidence of cathepsins. Assumption 2: Represented by dashed lines, the instrumental variables are not associated with any potential confounders. Assumption 3: The instrumental variables affect the outcome solely through the exposure, without any involvement in other causal pathways.

To investigate the causal link between cathepsins and CVDs, we used a two-sample MR research. Figure 1 depicts the research procedure.

### Instrumental variables selection

The SNPs selected for this study must meet the three core criteria of Mendelian Randomization (MR): 1. Correlation Hypothesis: Each genetic variation must exhibit a strong association with the exposure. 2. Independence Hypothesis: The genetic variation should be independent of both known and unknown confounders. 3. Exclusion Hypothesis: The genetic variation must influence the outcome solely through its impact on the exposure.

To ensure compliance with these assumptions, we established specific inclusion criteria: The SNPs incorporated into our analysis were selected based on their high correlation, with a significance threshold (*p* < 5 × 10^∧^−6) across the entire genome. Additionally, all included SNPs must be in linkage equilibrium, defined as a linkage distance of 10,000 kb and an r^∧^2 < 0.001.

The significance of regression analysis results was tested using the F-statistic, calculated as follows: F = R^2^ × (N - k - 1)/[(1 - R^2^) × k], where N is the sample size of GWAS for the cathepsins, k represents the number of SNPs, and R^2^ is the proportion of cathepsins status explained by each SNP. R^2^ is specifically calculated as R^2^ = 2 × beta^2^ × (1 - EAF) × EAF, where beta^2^ is the estimate of the genetic effect of each SNP on cathepsins, and EAF is the frequency of the effect allele. An F-value greater than 10 indicates that the included instrumental variables are strongly correlated with exposure.

### SNPs associated with cathepsins

Cathepsin-related SNPs were obtained from the INTERVAL study, which included 3,301 European individuals. All participants provided informed consent, and the INTERVAL study was approved by the National Research Ethics Service (approval number 11/EE/0538). Summary data from this study are available at [https://gwas.mrcieu.ac.uk] (Sun et al., 2018). All included cathepsin-related SNPs demonstrated a *p*-value <5 × 10^∧^-6, with no linkage disequilibrium observed between them (distance = 10,000 kb, r^∧^2 < 0.001). Detailed information about the included studies can be found in Supplementary Table S1.

### SNPs associated with CVDs

Our study included a variety of CVDs: coronary artery disease (CAD), MI, AF, CHF, aortic aneurysm (AA), pulmonary embolism (PE), and IS. Detailed outcome data are presented in Table 1.

**TABLE 1:** Basic information about the data sets included in the study.

Outcomes	Consortium	Sample size (cases/controls)	Population
Coronary artery disease	Mbatchou J et al.	352063	European
Myocardial infarction	Sakaue S et al.	20917/440906	European
Atrial fibrillation	Nielsen JB et al.	60620/970216	European
Chronic heart failure	Sakaue S et al.	14262/471898	European
Aortic aneurysm	Sakaue S et al.	3230/475964	European
Pulmonary embolism	Mbatchou J et al.	407746	European
Ischemic stroke	Sakaue S et al.	11929/472192	European

For CAD, SNPs were obtained from a study by Mbatchou J et al., involving 352,063 individuals (Mbatchou et al., 2021). MI-related SNPs were sourced from a study by Sakaue S et al., which included 20,917 MI patients and 440,906 controls (Sakaue et al., 2021). SNPs associated with AF were taken from a study by Nielsen JB et al., encompassing 60,620 AF patients and 970,216 controls (Nielsen et al., 2018). CHF-related SNPs were also obtained from Sakaue S et al., involving 14,262 CHF patients and 471,898 controls (Sakaue et al., 2021). For AA, the SNPs came from a study by Sakaue S et al., including 3,230 AA patients and 475,964 controls (Sakaue et al., 2021). PE-related SNPs were obtained from a study by Mbatchou J et al., with a total of 407,746 participants (Mbatchou et al., 2021). Lastly, IS-related SNPs were sourced from another study by Sakaue S et al., involving 11,929 IS patients and 472,192 controls (Sakaue et al., 2021). More details can be found in Supplementary Material.

### Mendelian randomization analysis

To determine if there is a causal relationship between cathepsins and CVDs, we used five methods, IVW, the weighted median, MR-Egger, the simple mode, and the weighted mode for the analysis. We primarily employed the IVW approach for MR analysis, which combines the effect sizes of individual SNPs to provide a weighted average estimate. This method is reliable when all SNPs are valid instruments and are not correlated (Xue et al., 2021). Additionally, we used the weighted median method, which is robust even when up to 50% of the SNPs are invalid instruments (Bowden et al., 2016). MR-Egger regression was used to assess and adjust for potential horizontal pleiotropy, where genetic variants affect the outcome through pathways other than the exposure (Bowden et al., 2015).

All data analyses were conducted using the TwosampleMR package in R software. The strength of the association was evaluated using OR, where an OR greater than 1 indicated that the exposure was a risk factor for the outcome, an OR less than 1 indicated that the exposure was a protective factor, and an OR equal to 0 indicated no effect. We also performed Reverse Mendelian Randomization analysis on positive findings to check for reverse causation.

### Sensitivity analysis

To assess horizontal pleiotropy, we utilized the MR-Egger analysis intercept. A significant intercept (*p* > 0.05) indicates the absence of horizontal pleiotropy. Additionally, we employed the MR-PRESSO method to further investigate horizontal pleiotropy by removing outliers from the data (Burgess et al., 2017).

We also conducted Cochran’s Q test to detect heterogeneity within our study’s results. A *p*-value greater than 0.05 suggested the absence of heterogeneity. To evaluate potential bias in the MR estimates due to any single genetic variation, we implemented the leave-one-out approach. The Leave-One-Out analysis in MR is a method for evaluating the stability of genetic instruments like SNPs. It involves removing each SNP one by one and reanalyzing the data to observe changes in the causal effect estimates. This technique helps identify influential SNPs, tests the robustness of results, and enhances transparency and credibility of the findings. Overall, it ensures that the causal inferences drawn from the MR studies are reliable and robust against individual genetic variations. We also reviewed the phenotypes database for secondary phenotypes of SNPs included in our study, excluding those associated with the outcome data. In addition, we utilized the ‘mRnd’ tool to assess the statistical power of our current MR analysis.

## Results

### Results of the two-sample Mendelian randomization analysis

**FIGURE 2:**
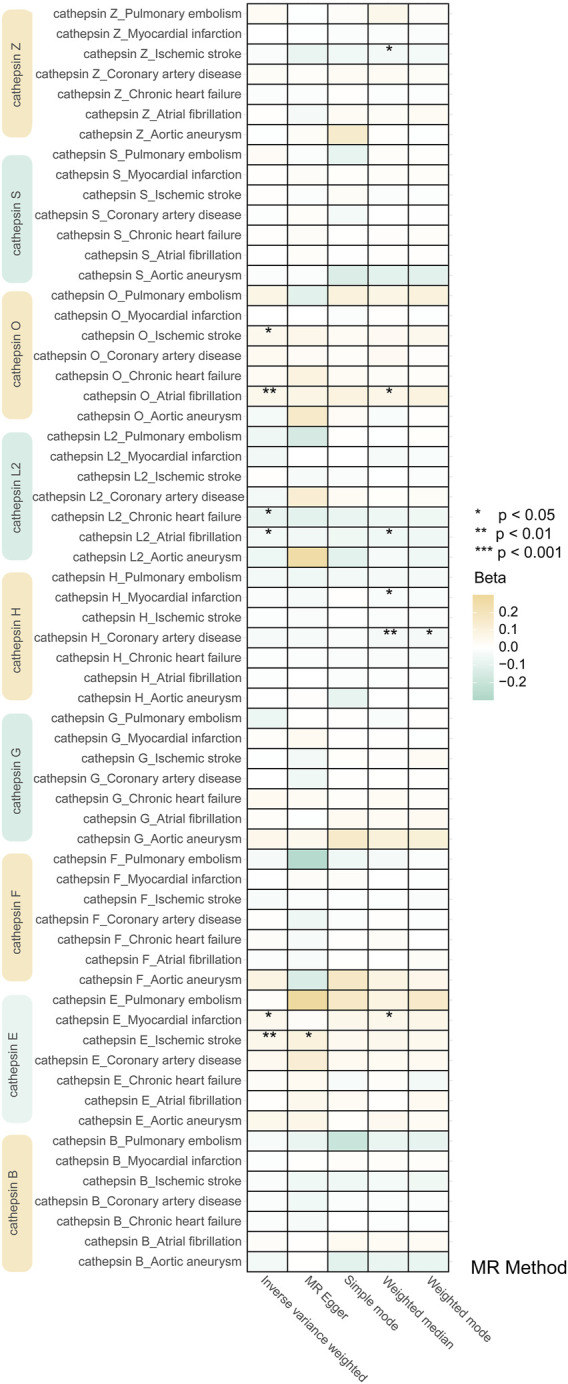
Heat map obtained using 5 MR detection methods of the effects of cathepsins on CVDs reveals that cathepsin E is a risk factor for MI and IS, cathepsin L2 offers protective effects against CHF and AF, and cathepsin O increases the risk for IS and AF. In the heatmap, yellow represents positive causal effects, while green represents negative causal effects. The darker the color, the stronger the causal effects. *indicates statistically significance.

**FIGURE 3:**
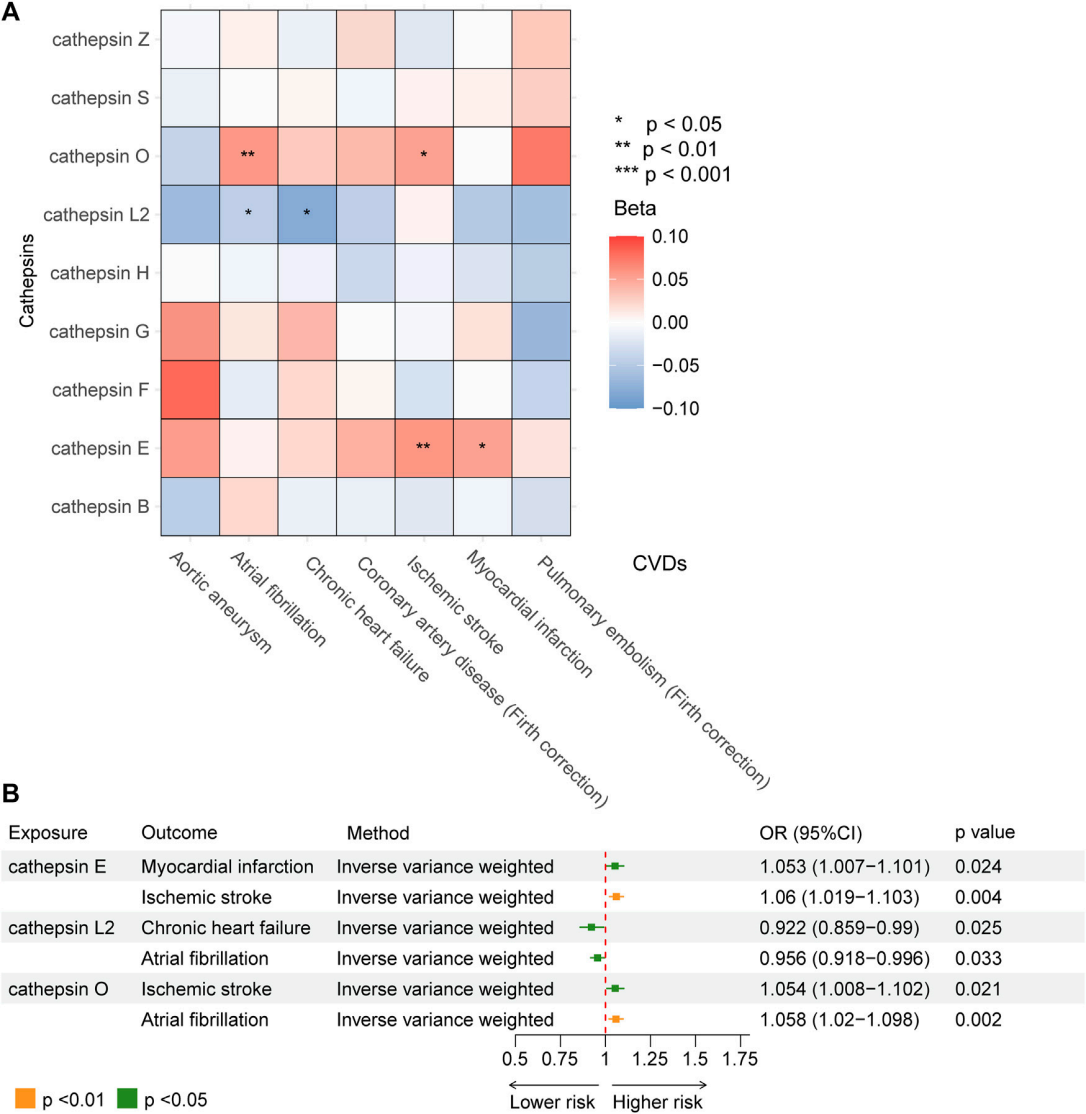
MR analysis demonstrating the causal relationship between cathepsins and seven type of CVDs. **(A)** Heat map obtained using IVW detection method of the effects of cathepsin E, cathepsin L2, and cathepsin O on CVDs. **(B)** Forest plot of the effects of cathepsin E, cathepsin L2, and cathepsin O on CVDs.

Our study investigated nine subtypes of cathepsins: B, E, F, G, H, L2, O, S, and Z, conducting MR analyses for each in relation to cardiovascular disease outcomes. The results, displayed in Figure 2. A heatmap depicting the causal effect (beta) of cathepsins on CVDs with five IVW methods, as detailed in Figure 3A. The findings revealed that cathepsin E may increase the risk of MI (OR = 1.053%, 95% CI: 1.007–1.101, *p* = 0.024) and IS (OR = 1.06%, 95% CI: 1.019–1.103, *p* = 0.004). Conversely, cathepsin L2 appears to reduce the risk of CHF (OR = 0.922%, 95% CI: 0.859–0.99, *p* = 0.025) and AF (OR = 0.956%, 95% CI: 0.918–0.996, *p* = 0.033). Cathepsin O was associated with an increased risk of IS (OR = 1.054%, 95% CI: 1.008–1.102, *p* = 0.021) and AF (OR = 1.058%, 95% CI: 1.02–1.098, *p* = 0.002), as detailed in Figure 3B. The other cathepsin subtypes did not show statistically significant effects on CVDs.

**FIGURE 4:**
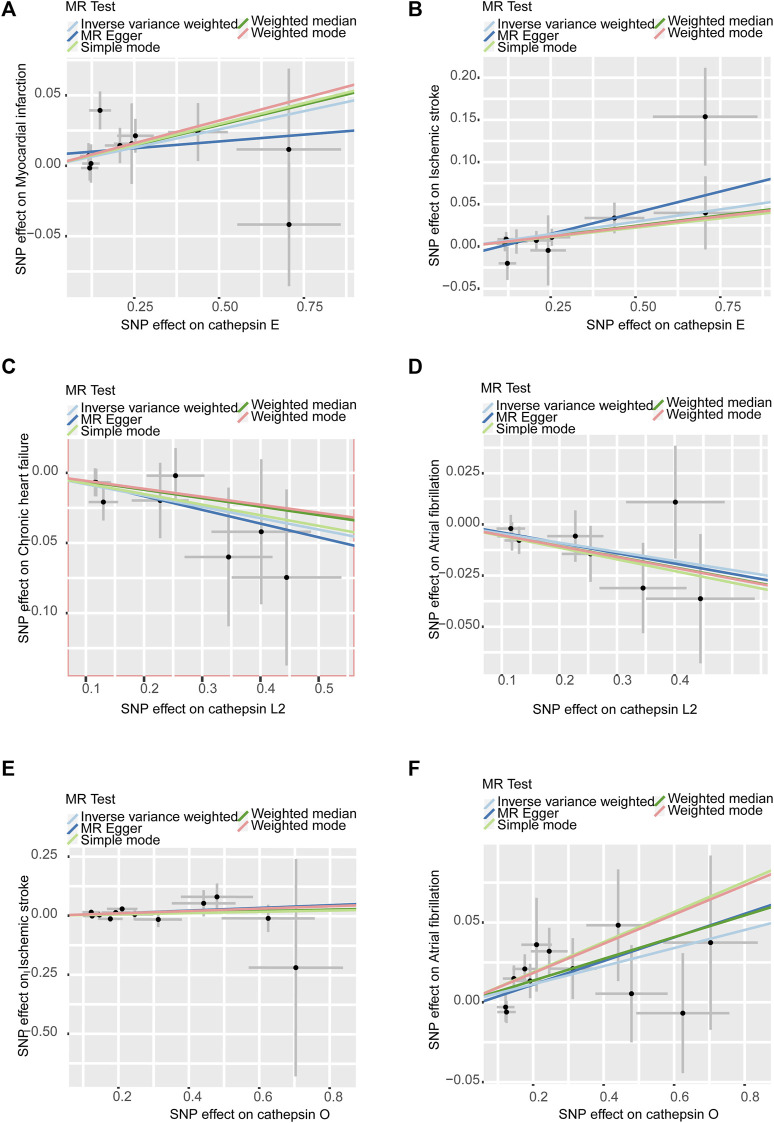
Scatter plots of sensitivity analysis: **(A)**. Cathepsin E and MI **(B)**. Cathepsin E and IS **(C)**. Cathepsin L2 and CHF **(D)**. Cathepsin L2 and AF **(E)**. Cathepsin O and IS **(F)**. Cathepsin O and AF. All assays showed no horizontal pleiotropy in MR analysis between cathepsins and CVDs.

**FIGURE 5:**
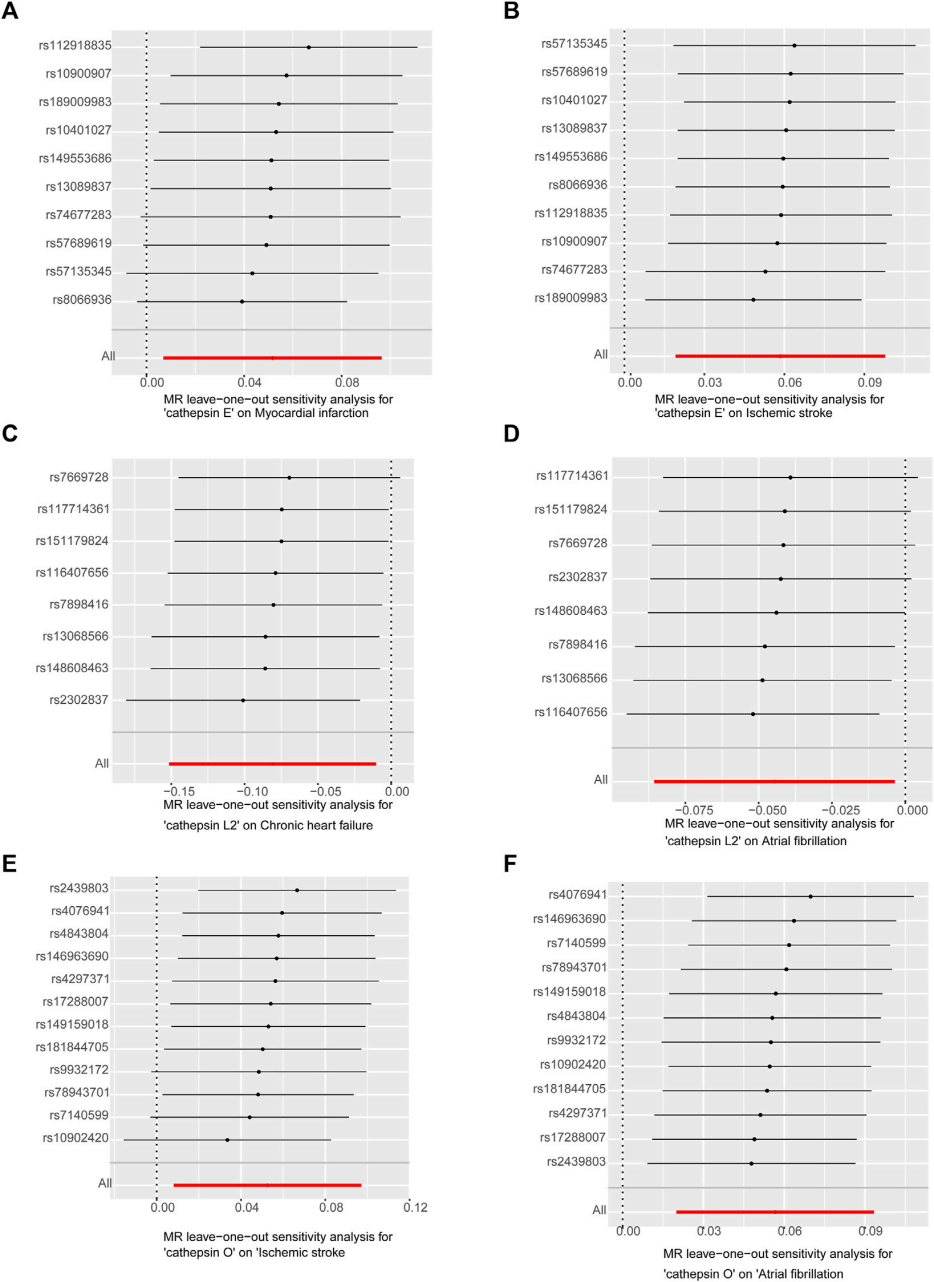
Leave-one-out analysis of sensitivity analysis: **(A)**. Cathepsin E and MI **(B)**. Cathepsin E and IS **(C)**. Cathepsin L2 and CHF **(D)**. Cathepsin L2 and AF **(E)**. Cathepsin O and IS **(F)**. Cathepsin O and AF. Leave-one-out analysis suggests stable and unbiased results.

In sensitivity analyses, the MR-PRESSO test detected no outliers, and both MR-Egger and MR-PRESSO tests were applied to assess horizontal pleiotropy. The results from both tests suggested the absence of horizontal pleiotropy in our findings (*p* > 0.05 for both tests) as shown in Figure 4. No heterogeneity was observed in the MR effect estimates according to Cochran’s Q test (*p* > 0.05). Details of the sensitivity analysis are presented in Table 2. According to the leave-one-out method (Figure 5), the removal of a single SNP did not impact the overall outcomes. The statistical power of our MR analysis was shown in the Table 3.

**TABLE 2:** Results of sensitivity analysis and heterogeneity test.

Exposures	Outcomes	Cochran’s Q statistic	*p*-value for Cochran’s Q	*p*-value for intercept	MR-PRESSO global test
Cathepsin E	MI	10.308	0.326	0.445	0.386
Cathepsin E	IS	6.640	0.674	0.270	0.729
Cathepsin L2	CHF	2.468	0.929	0.847	0.925
Cathepsin L2	AF	2.357	0.937	0.900	0.931
Cathepsin O	IS	10.99	0.443	0.914	0.456
Cathepsin O	AF	11.353	0.414	0.671	0.424

**TABLE 3:** The result of statistical power of MR analysis.

Exposure	Outcome	Method	Power
cathepsin E	Ischemic stroke	IVW	0.41
cathepsin E	Myocardial infarction	IVW	0.52
cathepsin L2	Atrial fibrillation	IVW	0.85
cathepsin L2	Chronic heart failure	IVW	0.75
cathepsin O	Atrial fibrillation	IVW	0.99
cathepsin O	Ischemic stroke	IVW	0.42

### Reverse Mendelian randomization analysis

**FIGURE 6:**
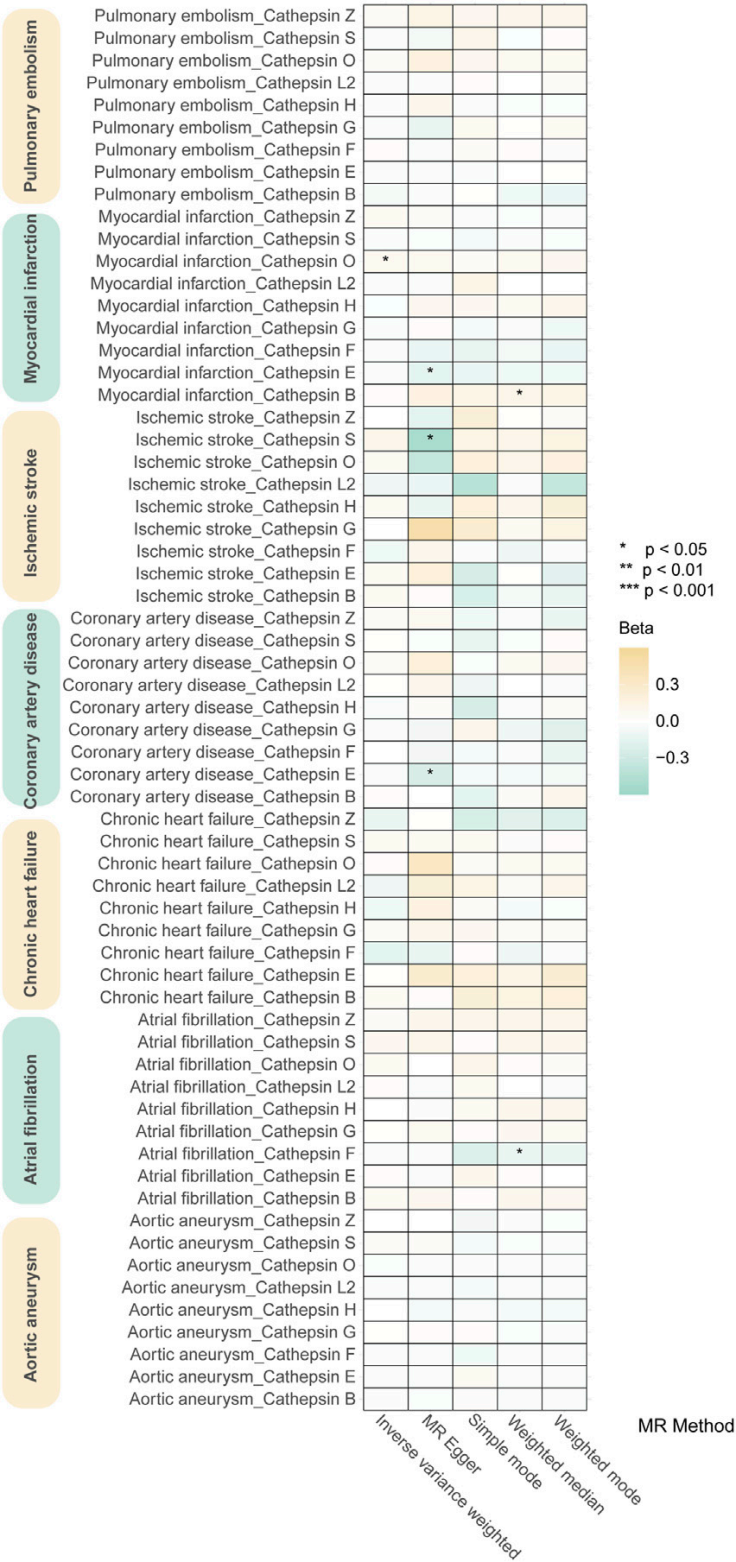
Heat map of the effects of CVDs on cathepsins reveals that there is no reverse causality between CVDs and cathepsins.

We employed reverse MR to investigate potential reverse causality between cathepsins and CVDs. The results of the IVW analyses indicated no causal relationship between MI, IS and cathepsin E; no causal relationship between CHF, AF and cathepsin L2; and no causal relationship between IS, AF and cathepsin O. The details of these MR analyses are illustrated in Figure 6.

## Discussion

The onset and progression of cardiovascular disease is a complex and lengthy process intertwined with a variety of risk factors, such as age, race, concurrent metabolic disorders (Hui et al., 2023), some dependent on protein metabolism (Shi et al., 1992). Cathepsins play a pivotal role in protein metabolism and are involved in the pathogenesis of CVDs by influencing the remodeling of extracellular matrix proteins (ECM). The ECM, primarily composed of collagen and elastin, is essential for maintaining the structural integrity of the cardiovascular wall. ECM remodeling is a key mechanism underlying CVDs, where cardiomyocytes and CVD-associated inflammatory cells (e.g., macrophages, leukocytes, and neutrophils) produce a significant amount of protein hydrolytic enzymes that degrade the ECM, thus contributing to the development of cardiovascular disease (Nagase et al., 2006; van Hinsbergh et al., 2006; Wu et al., 2018). However, most prior studies have been small cohort studies with limited sample sizes and low evidence levels. We propose using the MR method to explore the causal relationship between cathepsins and CVDs at the genetic level.

Cathepsins are a group of proteases that have been implicated in many immune diseases such as idiopathic myositis, interstitial lung disease (Cao et al., 2022), but there are no studies have investigated the causal relationship between cathepsins and CVDs, and ours is the first to explore this using the MR method. To date, fifteen human cathepsin proteases have been identified: A, B, C, D, E, F, G, H, K, L, O, S, L2, W, and Z. Based on their active site properties and catalytic mechanisms, they are primarily classified into three types: Cysteine Cathepsins, Serine Cathepsins, and Aspartic Cathepsins (Patel et al., 2018). Cathepsins are initially synthesized as preproenzymes. As they transit to the endoplasmic reticulum, the prepeptide is removed, forming procathepsin. The active forms of cathepsins are then generated in the acidic environments of late endosomes or lysosomes through proteolytic cleavage of the propeptide (Shi et al., 1992). Under physiological conditions, cathepsins reside in the lysosome, but exogenous oxidants can cause the lysosome to leak, leading to the release of cathepsin into the cytoplasm (Chwieralski et al., 2006). Cathepsin-mediated ECM degradation can destabilize arterial walls, potentially leading to the rupture of large and microvessels and the production of bioactive fragments that may be either harmful or beneficial to blood vessels (Felbor et al., 2000). Our study findings indicate that cathepsin E may increase the risk of MI and IS, while cathepsin L2 decreases the risk of CHF and AF, and cathepsin O increases the risk of IS and AF.

Cathepsin E, an endoprotease from the aspartic protease family, is most active in acidic environments but also retains activity at neutral pH. It is expressed in various tissues, particularly in immune system cells such as macrophages and dendritic cells (Chlabicz et al., 2011). The impact of Cathepsin E on MI and IS is mainly achieved through the following three mechanisms: First, Inflammatory Modulation: Cathepsin E may exacerbate vascular inflammation, which is a critical component of atherosclerosis progression. By activating inflammatory mediators, cathepsin E could contribute to endothelial dysfunction leading to MI and IS (Sukhova et al., 2003). Second, Extracellular Matrix Degradation: Cathepsin E degrades the ECM, and its breakdown products activate NLRP3 inflammatory vesicles, might facilitate the thinning of fibrous caps in atherosclerotic plaques, increasing the risk of plaque rupture that can lead to MI and IS (de Haan et al., 2013). Third, cathepsin E degrades low-density lipoproteins (LDL-P) and impedes cholesterol efflux from macrophages, contributing to the formation of foam cells. This process intensifies vascular atherosclerosis, a common pathogenic mechanism for MI and IS. Thus, cathepsin E may increase the risk of MI and IS by promoting atherosclerosis (Lutgens et al., 2007).

Cathepsin L2 (also known as cathepsin V) is a cysteine protease predominantly expressed in human keratinocytes and thymus, with peak activity in acidic environments (Lecaille et al., 2022). In 2002, Stypmann J et al. observed in animal experiments that a deficiency of cathepsin L (a homolog of human cathepsin L2) in mice led to structural cardiac degeneration and increased myocardial fibrosis (Stypmann et al., 2002). Subsequent studies indicated that cathepsin L expression in cardiomyocytes could inhibit cardiac remodeling and enhance cardiac function in mice by interfering with the AKT/GSK-3 beta signaling pathway (Tang et al., 2009). Improvements in the structural function of the heart, particularly the left atrium, have been shown to significantly reduce the incidence of AF. Given the homology between cathepsin L and cathepsin L2, it is speculated that cathepsin L2 may similarly act as a protective factor against CHF and AF. Further supporting this hypothesis, a 2017 study by Huang K et al. demonstrated that exogenous cathepsin L2 decreased the expression of hypertrophy markers in cardiomyocytes induced by angiotensin II (AngⅡ), effectively inhibiting AngII-induced increases in atrial natriuretic peptide (ANP), brain natriuretic peptide (BNP), and other substances, thus improving cardiac remodeling (Huang et al., 2017). Our study aligns with these findings, suggesting that cathepsin L2 may serve as a cardioprotective factor. We hypothesise that cathepsin L2 protects mainly through the following mechanisms: First, It likely inhibits Ang II activity through the AKT/GSK-3 beta pathway, improving cardiac remodeling and reducing the incidence of arrhythmias in AF (Lutgens et al., 2007). Second, Cathepsin L2 may possess antifibrotic properties that could be protective against cardiac remodeling processes associated with congestive heart failure and atrial fibrillation. By modulating the turnover of extracellular matrix proteins, cathepsin L2 could help maintain cardiac structure and function, thereby reducing the propensity for CHF and AF (Sun et al., 2011).

Cathepsin O, a member of the cysteine-type proteases, features a key cysteine residue at its active site. Like most cysteine proteases, cathepsin O is most active in acidic environments. Compared to other, more extensively studied cysteine proteases such as Cathepsin B, K, and L, research on cathepsin O is relatively sparse. Its specific functions within organisms and its potential clinical applications are areas that require further exploration. Current research primarily links cathepsin O to macrophage metabolism and macrophage-mediated extracellular matrix remodeling, yet its specific metabolic pathways and mechanisms remain to be fully elucidated (Shi et al., 1995). Our study is the first to explore at the genetic level the possibility that cathepsin O may increase the risk of stroke in the context of AF, although the connection to cathepsin O-mediated macrophage metabolism still requires confirmation through more detailed studies. We hypothesise that cathepsin O causes disease primarily through its effects on vascular and myocardial integrity. Its contribution to excessive matrix degradation or its effect on inflammation may predispose individuals to ischaemic events and arrhythmias (Cheng et al., 2011). There were also several significant limitations in our study. First, our study currently lacks experimental validation. It is crucial to note that the associations between cathepsin and CVDs must be confirmed through subsequent functional studies. Second, our two-sample MR analyses were conducted primarily using populations of European ancestry. Therefore, the generalizability of our findings to non-European populations and diverse ethnicities may be limited.

In conclusion, our study utilized the MR method to investigate the causal relationship between cathepsins and CVDs. The findings revealed that cathepsin E and cathepsin O are risk factors for CVDs, while cathepsin L2 serves as a protective factor. Our results are partially consistent with previous research, highlighting the established connections between cathepsins and CVDs. However, studies in this field remain limited, and further research is necessary to confirm and expand upon our findings. In addition, we tested and calculated the power values of the results of the MR analyses, in which the causal effect of cathepsin E on IS and MI, the effect of cathepsin L2 on the causality of CHF and the effect of cathepsin O on the causality of IS were less than 80%, and we have a cautious recommendation for the final test results.

## Data Availability

The original contributions presented in the study are included in the article/Supplementary Material, further inquiries can be directed to the corresponding author.
